# Eleven years of experience with the neurologic complications in Korean patients with acute aortic dissection: a retrospective study

**DOI:** 10.1186/1471-2377-13-46

**Published:** 2013-05-21

**Authors:** Seung-Jae Lee, Jae-Hyun Kim, Chan-Young Na, Sam-Sae Oh, Yang-Min Kim, Chang-Keun Lee, Dal-Soo Lim

**Affiliations:** 1 Department of Neurology, Sejong General Hospital, Bucheon, South Korea; 2 Department of Thoracic and Cardiovascular Surgery, Sejong General Hospital, Bucheon, South Korea; 3 Department of Radiology, Sejong General Hospital, Bucheon, South Korea; 4 Department of Cardiology, Sejong General Hospital, Bucheon, South Korea

## Abstract

**Background:**

This study attempts to explore the clinical features, possible mechanisms and prognosis of the neurologic complications in patients with acute aortic dissection (AD).

**Methods:**

Medical records of 278 consecutive patients with AD (165 with type A and 113 with type B dissection) over 11.5 years were retrospectively analyzed for clinical history, CT findings, neurologic complications and outcome. Neurologic complications were classified into early-onset or delayed-onset complications. Independent *t*-test or Chi-square test (or Fisher exact test) was used for comparing the different groups. Multivariable logistic regression analysis was performed to determine the independent association between variables.

**Results:**

The mean age of the included patients (145 male and 133 female) was 59.4 years (range 19–91 years). 41 patients (14.7%) had a neurologic complication, which included 21 with early-onset complication and 23 with delayed-onset complication, including 3 with both. Advanced age and classic type of dissection were independently associated with the neurologic complication in patients with type A dissection. The most frequent manifestation was ischemic stroke (26 patients, 9.4%), followed by hypoxic encephalopathy (9, 3.2%), ischemic neuropathy (5, 1.8%), spinal cord ischemia (5, 1.8%), seizure (2, 0.7%), hoarseness (1, 0.4%) and septic encephalopathy (1, 0.4%). Overall in-hospital mortality was 10.1%, whereas the complicated group had a mortality rate of 43.9%. Renal impairment, pulse deficit, neurologic complication and nonsurgical treatment were independent variables for determining in-hospital mortality in patients with type A dissection.

**Conclusions:**

The dominance of neurologic symptom in the early stage of AD may make its early diagnosis difficult. Besides chest pain and widened mediastinum in chest x-ray, variable neurologic symptoms including left hemiparesis with asymmetric pulse and hypotension may suggest underlying AD.

## Background

Acute aortic dissection (AD) is one of the lethal cardiac diseases involving the aorta. Although pain is a typical symptom, other various symptoms can be presented by the occlusive dissection of aortic branches, aneurysmal expansion of dissected aorta or hypotension related to hemopericardium [1]. Particularly, neurologic symptoms are not only frequent but often are dramatic and may mask the underlying disease. Therefore, it is crucial to understand the neurologic symptoms related to AD and to maintain a high-index of clinical suspicion in susceptible patients.

In this study, we attempt to explore the clinical features, possible mechanisms and prognosis of the neurologic complications in patients with acute aortic dissection (AD).

## Methods

The present authors initially studied 289 consecutive AD patients, who were admitted to the Cardiovascular Center at Sejong General Hospital within 14 days of symptom onset between January 2000 and September 2011. We reviewed retrospectively detailed clinical information including medical history, operation records, contrast-enhanced CT, echocardiography, in-hospital course and outcome including mortality. The study protocol was reviewed and approved by the institutional review board of Sejong General Hospital.

Of 289 patients initially studied, 5 patients with traumatic AD and 6 patients with incomplete study or hospital transfer were excluded. Finally, 278 patients were included for the present analysis. Two radiologists (Yang-Min Kim, Chang-Keun Lee), blinded to patient’s clinical data, analyzed dissection patterns in the aorta and its branches including innominate (IA), common carotid (CCA), subclavian (SA), celiac, superior mesenteric, renal and iliac arteries.

**Figure 1:**
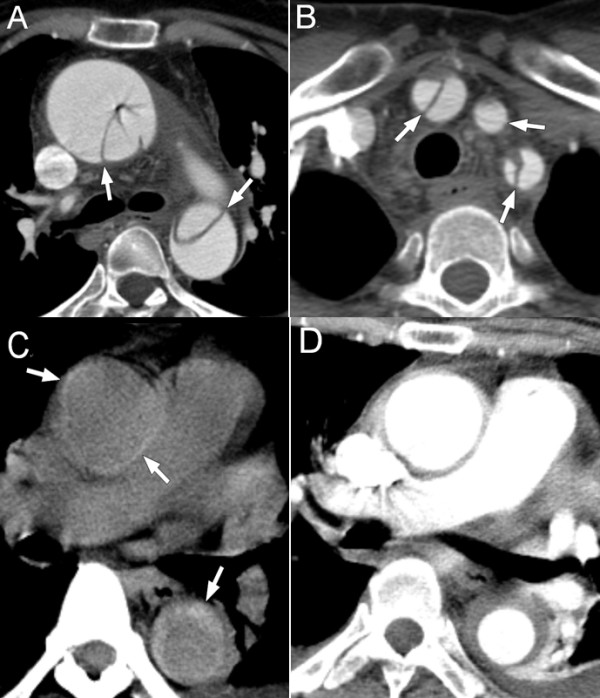
**Dissection type.** (**A**) Classic type A aortic dissection with visible intimal flap (arrow) inside dilated ascending and descending aorta. (**B**) Dissected supra-aortic branches (innominate, left common carotid and left subclavian artery) in the same patient as in figure A. (**C**) Type A intramural hematoma with crescent or circular high attenuation (arrow) in ascending and descending aortic wall on noncontrast CT. (**D**) No contrast enhancement of the thickened arotic wall in the same patient as in figure C.

We categorized the AD according to the Stanford (type A and B) and DeBakey (type I, II and III) classification [1]. In addition, as illustrated in the Figure 1, a typical double channel aorta with a visible intimal flap was regarded as the classic type of AD, and regional thickening of the aortic wall with a crescentic or circular high-attenuation area for precontrast CT and no contrast enhancement of the thickened aortic wall for contrast CT were diagnostic features of intramural hematoma (IMH) [2].

Neurologic complications were categorized into early- or delayed-onset complication. Early-onset complications included neurologic symptoms presented at the admission to our institution, and delayed-onset complication was defined as neurological disorders which were developed during medical or surgical treatment for two months after the admission. In addition, systolic hypotension was defined as systolic blood pressure below 100 mmHg at the admission.

According to the classification described in previous studies [3], neurologic complications were classified into different groups: 1) persistent or transient ischemic stroke, 2) hypoxic encephalopathy, 3) ischemic neuropathy, 4) spinal cord ischemia, 5) aneurysmal pressure phenomena and 6) others. 6-month follow-up data were collected from medical records.

We excluded postoperative delirium from neurologic complications because it was nonspecific, common, short-lasting confusional state which often happens in other surgical conditions. In addition, hypoxic encephalopathy was determined when sustained altered mentality was accompanied with an evidence specific to global CNS hypoperfusion.

Statistical analyses were performed with SPSS software, version 18.0. (SPSS Inc., Chicago, IL). Independent *t*-test or Chi-square test (or Fisher exact test) was used for comparing the different groups. In addition, multivariable logistic regression analysis was performed to determine the independent predictors for neurologic complications and in-hospital mortality in patients with type A dissection. Odds ratio (OR) and 95% confidential interval (CI) were obtained, and *P–*values < 0.05 were considered statistically significant.

## Results

### General characteristics of study population

The mean age at admission of 278 patients (145 male and 133 female) with AD was 59.4 years (range 19–91 years), and the most common predisposing conditions were hypertension (82.7%) and smoking (25.2%). There were 165 type A and 113 type B dissections. Among 165 patients with type A dissection, 40 patients were medically treated, whereas 121 patients underwent replacement of the aorta, including 87 cases of supracommissural replacement of the ascending aorta and 34 cases of composite replacement of the aortic valve and ascending aorta. Furthermore, 2 patients received pericardiostomy alone and the other two underwent thoracic endovascular aortic repair.

A hundred out of 113 patients with type B dissection received medical intervention, and the other 13 patients received operative procedures, which included 8 cases of aortic graft replacement, 3 cases of aorto-bi-iliac graft replacement, one case of femorofemoral bypass and one case of right axillary-bifemoral bypass.

As shown in Table 1, patients with type A dissection showed a higher prevalence of female sex, syncope, widened mediastinum, any pulse deficit and the involvement of aortic branch vessels, with a greater frequency of systolic hypotension, neurologic symptoms and in-hospital mortality. Compared with patients with type A dissection, patients with type B dissection showed a higher prevalence of current smoking and IMH. Among patients with type A dissection, 126 patients (76.4%) had DeBakey type I dissection, and 39 patients (23.6%) had type II dissection.

**Table 1:** General characteristics of patients with acute aortic dissection: mean ± SD, n (%)

	**Type A**	**Type B**	***P***
	**n = 165**	**n = 113**	
Age	59.2 ± 14.3	59.8 ± 13.9	0.742
Male	75 (45.5)	70 (61.9)	0.007
Hypertension	132 (80.0)	98 (86.7)	0.145
Diabetes mellitus	13 (7.9)	11 (9.7)	0.588
Hyperlipidemia	21 (12.7)	16 (14.2)	0.730
Current smoking	32 (19.4)	38 (33.6)	0.007
Previous ischemic stroke	17 (10.3)	9 (8.0)	0.511
Ischemic heart disease	16 (9.7)	12 (10.6)	0.802
Atrial fibrillation	8 (4.8)	6 (5.3)	0.863
Marfan syndrome	16 (9.7)	7 (6.2)	0.298
Systolic hypotension	38 (23.0)	4 (3.5)	< 0.001
Syncope	23 (13.9)	5 (4.4)	0.014
Widened mediastinum	101 (61.2)	45 (39.8)	0.001
Pain	140 (84.8)	103 (91.2)	0.120
azotemia (creatinine > 2 mg/dL)	23 (13.9)	13 (11.5)	0.553
Mesentery ischemia	6 (3.6)	1 (0.9)	0.247
Any pulse deficit	12 (7.3)	1 (0.9)	0.017
Intramural hematoma	43 (26.1)	50 (44.2)	0.002
Any neurologic symptom	36 (21.8)	5 (4.4)	< 0.001
Aortic branch involvement	95 (57.6)	49 (43.4)	0.020
In-hospital mortality	26 (15.8)	2 (1.8)	< 0.001

### Characteristics of patients with neurologic complication

Among 278 patients, 41 patients (14.7%) had neurologic symptoms, consisting of 36 cases of type A dissection and 5 cases of type B dissection. Out of the 41 patients, 21 had an early-onset neurologic complication and 23 had a delayed-onset neurologic complication; 3 patients presented both.

6 patients with type A dissection had a combination of different neurologic complications: hypoxic encephalopathy and hemiplegic stroke (twice), hypoxic encephalopathy with stroke and ischemic neuropathy (once), generalized seizure associated with hypoxic encephalopathy or hemiparetic stroke (twice), paraplegia and ischemic neuropathy (once). One patient with type B dissection had both posterior-circulation stroke and paraplegia (Table 2).

**Table 2:** Neurologic complications of patients with AD: n (%)

	**Type A**		**Type B**		**Total**
	**n = 36**		**n = 5**		**n = 41**
	**Early**	**Delayed**	**Early**	**Delayed**	
Ischemic stroke	8	14	0	4	26 (63.4)
Hypoxic encephalopathy	7	2	0	0	9 (22.0)
Ischemic neuropathy	4	1	0	0	5 (12.2)
Spinal cord ischemia	1	2	1	1	5 (12.2)
Vocal cord paralysis	1	0	0	0	1 (2.4)
Seizure	2	0	0	0	2 (4.9)
Septic encephalopathy	0	1	0	0	1 (2.4)

*AD* Acute aortic dissection.

Table 3 shows the comparison between the neurologically-complicated and uncomplicated groups in patients with type A dissection. Between the two groups, there were no significant differences in the frequency of male, vascular risk factors, pain, aortic regurgitation, cardiac tamponade and supra-aortic involvement. The complicated group was older, and had a higher frequency of systolic hypotension and classic type dissection (*P* < 0.05).

**Table 3:** Comparison between the neurologically-complicated and uncomplicated groups in patients with type A dissection: mean ± SD, n (%)

	**Neurologic complication (+)**	**Neurologic complication (−)**	***P***
	**n = 36**	**n = 129**	
Age	65.6 ± 12.4	57.4 ± 14.3	0.002
Male	15 (41.7)	60 (46.5)	0.606
Hypertension	27 (75.0)	105 (81.4)	0.396
Diabetes mellitus	1 (2.8)	12 (9.3)	0.302
Hyperlipidemia	3 (8.3)	18 (14.0)	0.572
Valvular heart disease	4 (11.1)	6 (4.7)	0.229
Current smoking	4 (11.1)	28 (21.7)	0.232
Previous ischemic stroke	4 (11.1)	13 (10.1)	0.767
Ischemic heart disease	2 (5.6)	14 (10.9)	0.527
Atrial fibrillation	3 (8.3)	5 (3.9)	0.374
Pain	32 (88.9)	108 (83.7)	0.601
Systolic hypotension	15 (41.7)	23 (17.8)	0.003
Aortic valve regurgitation	14 (38.9)	53 (41.1)	0.812
Cardiac tamponade	14 (38.9)	30 (23.3)	0.061
Supra-aortic branch involvement	22 (61.1)	56 (43.4)	0.060
Classic type	32 (88.9)	90 (69.8)	0.030

Multivariable logistic regression using variables with *P* < 0.2 showed that age (*P* = 0.001; OR 1.064; CI 1.026-1.103) and classic type of dissection (*P* = 0.019; OR 4.625; CI 1.290-16.577) were independently associated with neurologic complication in patients with type A dissection.

### Detailed clinical features of AD-related neurologic complications

1) Ischemic stroke

As shown in Table 4, 26 (9.4%) patients experienced an ischemic stroke, which included 22 with type A and 4 with type B dissection. Among 22 patients with type A dissection, 8 patients including a case of TIA had an early-onset stroke and 14 patients had a delayed-onset stroke (9 within 14 days of surgery, 3 between15 and 60 days after surgery and the other 2 during medical treatment). 4 patients with type B dissection had a delayed–onset stroke (1 on the 17th postoperative day and the other 3 during medical treatment).

Early-onset stroke was all referable to the anterior circulation, predominantly right-sided (87.5%). One or more main branches of the aortic arch were involved in 6 out of 8 patients (75%) with early-onset stroke. IA was most frequently involved (75.0%), followed by right CCA, left CCA, and SA.

In contrast, delayed-onset stroke affected similarly bilateral carotid territories, and also included lesions in bilateral carotid, posterior-circulation and anterior/posterior-circulation territories. Among these patients, 9 (50%) had dissected supra-aortic branches. The most frequently dissected branches were IA and CCA.

Among the 26 patients, 8 patients (30.8%) expired within 6 months of the disease onset (3 cases from hemispheric stroke with brain herniation, 2 cases from aortic rupture, 2 cases from sepsis with multiple organ failure and a case from mesentery ischemia and renal failure). Additionally, 9 patients (34.6%) remained functionally dependent six months later.

2) Hypoxic encephalopathy

9 (3.2%) patients with type A dissection showed decreased mental function caused by the brain hypoxia. Supra-aortic branches were involved in 8 patients (88.9%), all in whom IA and CCA (of bilateral side in 7 cases and right side alone in 1 case) were involved by dissection. In addition, systolic hypotension related to cardiac tamponade was observed in 8 patients (88.9%).

Among the 9 patients, 7 patients presented this as the initial symptom. 6 out of the 7 patients expired within 3 weeks of the disease onset, and only one patient survived after surgery but remained totally dependent in activities of daily living as a result of right hemispheric stroke.

The other two patients did not regain consciousness after surgery, and finally expired within 3 weeks. Therefore, among 9 patients with hypoxic encephalopathy, 8 (88.9%) died within weeks of the disease onset.

3) Ischemic neuropathy

5 patients with type A dissection had an ischemic neuropathy (1.8%). At admission, 4 patients presented localized pain, paresthesia, pulse deficit with variable degrees of motor deficit in their legs, which could be attributed to the occlusion of the iliofemoral arteries because all their iliac arteries were involved in the dissection (bilateral side in 3 cases and left side alone in a case). Two patients recovered after surgery, while the other two died during hospitalization due to the accompanying hemispheric stroke and sepsis, respectively.

The other patient was found to have right arm paralysis, coldness and paresthesia immediately after operation. However, those symptoms were relieved at hospital discharge.

4) Spinal cord ischemia

5 patients (1.8%) developed acute paraplegia and sensory change below T8-10. 3 patients developed complete spinal cord infarct, whereas the other 2 patients suffered incomplete cord injury resulting in the anterior cord syndrome with relative sparing of the position and vibration sense.

Among the 5 patients, 2 patients presented the symptoms at admission. One patient with type A dissection expired on the 6th hospital day because of aortic rupture. The other patient with type B dissection remained wheelchair-bound 6 months later.

3 patients developed acute paraplegia postoperatively (1 on the first postoperative day and the other 2 between 50–60 days after surgery). Of the two patients with type A dissection, one died from a septic complication, and the other patient was paraplegic and incontinent at 6 months. One patient with type B dissection stayed locked-in 6 months later because of severe posterior-circulation infarct.

5) Aneurysmal pressure phenomenon

A patient with type A dissection presented hoarseness as an initial symptom, which could be attributable to the compression of recurrent laryngeal nerve against the aneurysmal dilatation of AD. After surgery, hoarseness of voice resolved within 4 weeks.

6) Others: seizure and septic encephalopathy

Two patients with type A dissection showed a seizure manifestation (loss of consciousness, convulsive movement and post-ictal confusion). One case was related to ischemic stroke, and the other was secondary to hypoxic encephalopathy causing in-hospital death. Besides, one patient with type A dissection developed encephalopathy induced by severe sepsis and pneumonia, which rendered her disease inoperable. Despite intensive medical care, she expired during hospitalization.

**Table 4:** Characteristics of stroke in AD patients: n (%)

	**Early-onset stroke**	**Delayed-onset stroke**	**Total**
	**n = 8**	**n = 18**	**n = 26**
Type A	8 (100)	14 (77.8)	22 (84.6)
Vascular territory of lesion			
Anterior circulation	8 (100)	13 (72.2)	21 (80.8)
Right carotid	7 (87.5)	6 (33.3)	13 (50.0)
Left carotid	1 (12.5)	4 (22.2)	5 (19.2)
Bilateral carotid	0 (0.0)	3 (16.7)	3 (11.5)
Posterior circulation	0 (0.0)	1 (5.6)	1 (3.8)
Anterior & posterior	0 (0.0)	4 (22.2)	4 (15.4)
Supra-aortic vessel involvement	6 (75.0)	9 (50.0)	15 (57.7)
Innominate artery	6 (75.0)	9 (50.0)	15 (57.7)
Common carotid artery	6 (75.0)	8 (44.4)	14 (53.8)
Right	5 (62.5)	5 (27.8)	10 (38.5)
Left	3 (37.5)	5 (27.8)	8 (30.8)
Subclavian artery	4 (50.0)	3 (16.7)	7 (26.9)
Right	2 (25.0)	1 (5.6)	3 (11.5)
Left	2 (25.0)	2 (11.1)	4 (15.4)
6-month MRS			
0-2	4 (50.0)	5 (27.8)	9 (34.6)
3-5	1 (12.5)	8(44.4)	9 (34.6)
6 (death)	3 (37.5)	5 (27.8)	8 (30.8)

*AD* acute aortic dissection, *MRS* modified Rankin scale.

### Statistical analysis for in-hospital mortality

Overall in-hospital mortality was 10.1% (15.8% for type A dissection and 1.8% for type B dissection), whereas patients with neurologic complication had a mortality rate of 43.9% (18 out of 41paitents). Because the in-hospital death of patients with type B dissection was rare as the rate of 1.8%, variables related to in-hospital mortality were analyzed for patients with type A dissection alone.

Among patients with type A dissection, in-hospital mortality rate was significantly higher in patients with early-onset (12 out of 20, 60% versus 14 out of 145, 9.7%; *P* < 0.001) or delayed-onset (8 out of 19, 42.1% versus 18 out of 146, 12.3%; *P* = 0.001) neurologic complication than patients without neurologic complication. The mortality group was older (67.7 ± 12.3 versus 57.6 ± 14.1; *P* = 0.001), and had a higher frequency of systolic hypotension (50.0% versus 28.0%; *P* < 0.001), syncope (30.8% versus 10.8%; *P* = 0.007), azotemia (50.0% versus 7.2%; *P* < 0.001), mesenteric ischemia (23.1% versus 0%; *P* < 0.001), pulse deficit (26.9% versus 3.6%; *P* = 0.001), nonsurgical treatment (57.7% versus 19.4%; *P* < 0.001) and neurologic complication (69.2% versus 12.9%; *P* < 0.001). Besides, in comparison with classic type, IMH type showed a statistical trend toward less mortality (3 out of 43, 7.0% versus 23 out of 122, 18.9%; *P* = 0.088).

Logistic regression analysis (using the variables of age, previous stroke, syncope, systolic hypotension, azotemia, pulse deficit, neurologic complication and nonsurgical treatment) demonstrated that azotemia (*P* = 0.009; OR 6.990; CI 1.629-29.994), pulse deficit (*P* = 0.023; OR 11.581; CI 1.397-95.997), neurologic complication (*P* = 0.001; OR 10.782; CI 2.653-43.813) and nonsurgical treatment (*P* = 0.002; OR 11.628; CI 2.435-55.523) were independent predictors for determining in-hospital mortality in patients with type A dissection.

## Discussion

Neurologic complications were manifested in 14.7% of all patients with AD, and 21. 8% of patients with type A dissection, which is approximately similar to the range between 17 and 48% reported by previous studies [3-6]. A wide variation in the prevalence between studies may be due to the failure to record neurological examinations in detail in critically ill patients leading to an underestimation of neurologic complications [3].

In accordance with previous reports, ischemic stroke was the most frequent neurologic manifestation [3,4,6]. Early-onset stroke tends to be more frequently hemispheric compared with vertebrobasilar territory and predominantly right-sided. The hemispheric predominance is accounted for the fact that the carotid artery origins are much more vulnerable to the advancing dissection due to their proximity to the aortic arch [3,4,6,7]. In addition, a right-side dominance of lesions can be explained by different mechanical dynamics in the progression of the dissecting hematoma. In other words, the hydraulic stress is the greatest in the right lateral wall of the ascending aorta [7-9]. This assumption can be supported by the right dominant distribution of supra-aortic branch dissections in our patients.

In contrast, 50% of patients with delayed-onset stroke and 25% of patients with early-onset stroke demonstrated no supra-aortic branch involvement. In particular, delayed-onset strokes affect both hemispheres similarly and were often bilateral. In addition, they included a case of posterior-circulation infarct. This suggests that ischemic stroke can be caused not only by the extension of dissection toward the supra-aortic vessels, but also by other mechanisms such as thromboembolism or severe hypotension especially in delayed period of the disease course. Furthermore, perioperative strokes might be related to various perioperative factors such as circulatory arrest, or microemboli during operation.

Occasionally, stroke symptoms may completely dominate the clinical picture in the early stage of AD, masking the underlying disease. Among our patients, two developed right hemispheric stroke with hemiplegia related to right CCA dissection without clear signs of AD. Because stroke symptoms seemed clinically serious, the accurate diagnosis was delayed in a nearby stroke center. After all, type A dissection was detected during catheter cerebral angiography, and after undergoing right CCA stenting, they were transferred to our hospital. Thus, the early diagnosis of AD may be difficult without a close clinical vigilance for the disease because this is a rare cause of ischemic stroke.

Moreover, AD is challenging in the decision-making of intravenous thrombolysis for patients with hyperacute ischemic stroke due to the difficulty to diagnose within a narrow time frame. A literature review revealed 8 cases treated with IV thrombolysis for acute ischemic stroke secondary to AD. 3 out of 4 patients treated with full-dose therapy died from hemorrhagic complication [6,10-12], while only one of 4 patients with partial-dose therapy died without surgery [13-16]. In the latter cases, infusion was discontinued immediately after the prompt diagnosis of AD. Thrombolysis is, therefore, supposed to decrease the probability of survival in patient with AD, although it has been reported to be feasible in patients with dissection solely of the internal carotid artery [17]. Especially when left hemiplegic infarct is present with asymmetric pulse and unexplained hypotension, AD with atypical presentation should be excluded [18]. In addition, bed-side carotid ultrasonography can be useful to ascertain concomitant CCA dissection [18,19].

AD-related hypoxic encephalopathy was fatal. Most of the patients (88.9%) expired during their hospital stay. The main mechanism was reduced cerebral perfusion, which derives from the extension of the dissection along the aorta occluding the origin of the supra-aortic vessels and severe hypotension caused by shock from cardiac tamponade.

Of 5 cases with AD-related ischemic neuropathy, 4 were associated with occlusive dissections of iliofemoral arteries, and the other was presumed to result from emboli to the subclavian artery during operation. Because AD-related ischemic neuropathy can be developed without chest or abdominal pain, benign diseases such as herniated disk and thoracic outlet syndrome should be initially differentiated [20,21]. Besides, postoperative compressive neuropathy may arise during general anesthesia of long duration. However, pulse deficit and development of other signs of limb ischemia narrow the differential diagnosis to AD and arterial embolus [21,22].

Acute paraplegia occurred in 1.8% of our patients, which was within the previously reported range of between 0% and 8% [3-6]. The injury level was mostly assumed to be mid-thoracic cord, the watershed zone between the territories of the artery of Adamkiewicz and the thoracic radicular artery. Occasionally, the injury spared posterior column function, presenting the anterior cord syndrome. This fact can be explained by the vascular anatomy of the spinal cord indicating that the anterior spinal artery supplying the anterior two-thirds of the cord has fewer collaterals than the posterior spinal arteries [7,21]. In addition to the anterior cord syndrome and complete cord infarct in our series, Brown-Sequard syndrome, progressive myelopathy and transient cord ischemia have also been described [3,7,23].

Pressure phenomena from the aneurysmal segment in AD have rarely been reported in literature. Symptoms have included dysphagia owing to esophageal compression [24], Horner’s syndrome resulting from pressure on the cervical sympathetic ganglion [6,7] and hoarseness caused by compression of the left recurrent laryngeal nerve [25]. In our study, one patient presented hoarseness.

Previous studies have discussed the association between neurologic complication and in-hospital mortality in patients with type A dissection [3,5,6,26-28]. In accordance with some previous studies, neurologic complication was independently associated with hospital death [26-28]. However, other recent data showed that neurologic complication had only statistical tendency or marginal significance toward the risk of in-hospital death [5,6]. This might be attributable to the difference of study population, operation factors (operator skill, surgical process, or anesthesia) or statistical variables between the studies.

In our study, IMH type accounted for 26.1% of our patients with type A dissection, and showed a significantly lower frequency of neurologic complication and a trend toward less hospital mortality, when compared with classic type AD. In addition, it was demonstrated, to the best of our knowledge for the first time, that the classic type of AD was independently associated with neurologic complication in patients with type A dissection. This is in close agreement with the results of previous Korean and Japan studies, which have shown that IMH is more commonly seen in far-eastern countries and have a tendency to present fewer mortality and neurologic signs than classic type AD or western-type IMH [29-32]. Thus, regional heterogeneity of IMH regarding the incidence and disease behavior might affect the results of our study.

The strength of our study lies in its large sample size, allowing relatively high statistical power. As a matter of fact, this is the first comprehensive data on the neurologic complication of Asian patients with AD, with the largest number of patients in a study on this issue to date. Moreover, differently from those previous studies [3,6,7], IMH, a variant form of AD, was discriminated from classic type AD in the data analysis. Therefore, the present authors expect that this report will lead to a deeper understanding of AD-related neurologic complications.

However, our study has some limitations. First, there may have been a selection bias because this study was based on data from a single center. Second, our study presents limitations associated with the nature of a retrospective study design using medical records. As previously stated, the frequency of neurologic complications may be underestimated because it appears that all patients with unstable vital signs were not properly examined by a neurologist. In addition, some neurologic symptoms classified as “early-onset complications” in this study might have been developed during the clinical management of other hospitals. Actually, 6 of 21 cases classified as belonging to the group with early-onset complication were ascertained to come to our hospital after a short-term care at local hospitals. In some of those cases, the exact time of symptom onset was unclear, and medical records of the transferring hospitals were usually unavailable at the time of study. Thus, “early-onset complication” was unavoidably defined as neurologic symptom presented at the admission to our institution.

## Conclusions

AD often causes neurologic complications, especially in patients with classic type A dissection. The most common manifestation was ischemic stroke and hypoxic encephalopathy. The presumed mechanisms were aortic branch dissection causing luminal occlusion, emboli from thrombosed vascular lumen and hypotension under the condition of AD. It is important to perceive them because the dominance of neurologic symptoms in the early stage of AD may make its early diagnosis difficult. Besides typical chest pain and widened mediastinum in chest x-ray, variable neurologic symptoms including left hemiparesis with asymmetric pulse and hypotension may suggest underlying AD.

## Competing interests

The authors declare that they have no competing interests.

## Authors’ contributions

All authors met the criteria for authorship and have approved the contents of the text. SJL and JHK contributed to study concept and design. SJL did statistical analysis, wrote the first draft and revised the manuscript. JHK, CYN, SSO and DSL participated in the acquisition of data. JHK, CYN, SSO, DSL, YMK and CKL contributed to the analysis and interpretation of data. All authors read and approved the final manuscript.

## Pre-publication history

The pre-publication history for this paper can be accessed here:

http://www.biomedcentral.com/1471-2377/13/46/prepub
